# Application value and controversies of artificial intelligence in public health prevention and control of postpartum depression

**DOI:** 10.3389/fpubh.2026.1812867

**Published:** 2026-05-19

**Authors:** Meng Xu, Jin-e Xu

**Affiliations:** Department of Obstetrics, Nanjing Drum Tower Hospital Group Suqian Hospital, Suqian, China

**Keywords:** applications, artificial intelligence, controversies, postpartum depression, public health

## 1 Introduction

Postpartum depression (PPD) is a major global public health concern for maternal and child health. According to the World Health Organization (WHO), approximately 10–20% of postpartum women globally are affected by PPD, with prevalence peaking at 25% in low- and middle-income countries (1). PPD exerts profound adverse effects on maternal, infant, and family wellbeing, and imposes substantial disease burden and socioeconomic costs worldwide. Traditional PPD screening relies on self-administered questionnaires, limited by notable shortcomings including temporal lag, low response rates, and excessive dependence on in-person clinical encounters (2, 3). For women with confirmed PPD, access to effective treatment remains hindered by multiple barriers: insufficient maternal and child health (MCH) resources limiting access to professional mental health services; inadequate standardized prevention and control capacity in primary care, leading to extremely poor service accessibility in remote regions; maternal reluctance to seek care due to mental illness-related stigma; and concerns regarding pharmacotherapy safety during breastfeeding.

In recent years, artificial intelligence (AI) has emerged as a transformative technology in mental health, driving a paradigm shift in PPD public health prevention and control (4, 5). The arguments and perspectives presented in this Opinion article are based on peer-reviewed empirical studies, systematic reviews, and international public health guidelines published up to 2025, with a focus on methodologically robust research in AI and maternal mental health. This article has three core objectives: (1) to elucidate the core application value of AI in reshaping PPD public health prevention and control paradigms; (2) to critically examine the key ethical controversies and real-world implementation barriers of AI in this field; and (3) to propose evidence-based principles for standardized AI application in PPD public health practice, to provide a theoretical reference for building an equitable and efficient maternal mental health prevention and control system.

## 2 Application of AI in PPD prevention and screening

A core value of AI is its ability to reconstruct the PPD public health screening system, and facilitate a paradigm shift from passive individual treatment to active population- level early warning (5). Traditional PPD screening, centered on the Edinburgh Postnatal Depression Scale (EPDS), is heavily dependent on maternal outpatient attendance and frontline healthcare workers' professional competency, resulting in substantial gaps in screening coverage and marked temporal delays (2, 3). AI addresses these limitations through three evidence-based core approaches:

(1) Enabling multimodal, non-invasive, full-cycle risk early warning. Leveraging large language models, AI can integrate prenatal electronic health records (EHRs), physiological metrics (e.g., heart rate variability, sleep architecture, activity levels) from wearable devices, and emotional features from maternal speech and text data, to enable early identification of PPD high-risk individuals and proactive intervention (6); Recent empirical data indicate that machine learning models integrating these multimodal data outperform conventional scale-based screening approaches for PPD risk (7).

(2) Substantially expanding population-level screening coverage in primary care. AI-assisted screening tools can be integrated into national MCH information platforms to automatically extract key risk factors (e.g., prior psychiatric history, pregnancy complications, inadequate social support) and achieve automated population-level risk stratification. This integration requires no additional hardware investment, making it feasible for implementation in low-resource settings through routine EHR data collected by community health workers.

(3) Mitigating disease stigma barriers via anonymized screening. Online AI-based screening eliminates the need for in-person consultations, significantly increasing maternal willingness to participate compared with traditional outpatient screening, and reducing screening delays and missed diagnoses due to mental illness-related stigma (8). Multicenter clinical data demonstrate that online AI-assisted screening tools exhibit significant efficacy in PPD risk assessment accuracy and tailoring individualized intervention strategies (9).

Unlike traditional screening tools, AI-driven detection can be implemented continuously without imposing additional burden on postpartum women or clinical systems, demonstrating favorable scalability in both resource-rich and resource-constrained healthcare settings.

## 3 Application of AI in PPD intervention

Another core application value of AI is its capacity to support the development of a tiered PPD intervention system, to achieve precise allocation and efficient utilization of public health resources. Traditional PPD intervention systems face severe resource mismatch: professional psychological and psychiatric resources are heavily concentrated in urban hospitals, while primary care MCH institutions lack standardized intervention capacity. This leaves women with mild-to-moderate PPD in a long-term management gap, and obstructs referral pathways for those with severe disease.

AI enables the establishment of a hierarchical intervention model and an integrated screening-intervention-follow-up-referral care continuum, through three key pathways:

First, delivering highly accessible, standardized interventions for women with mild-to-moderate PPD. Chatbots and virtual assistants trained in evidence-based modalities including mindfulness-based stress reduction (MBSR), cognitive behavioral therapy (CBT), and interpersonal therapy (IPT) can provide on-demand, low-risk mental health support, overcoming barriers related to time, geographic location, and ADDIN EN.CITE (7, 10) stigma. For low-resource settings, offline AI chatbot tools compatible with basic feature phones and lightweight modules integrated into existing maternal health SMS systems can be deployed to address internet and smartphone access barriers.

Second, enabling dynamic, precise allocation of public health resources. Based on regional PPD prevalence, risk factor distribution, and medical resource allocation, AI can generate data-driven insights for public health decision-makers, facilitating the expansion of MCH mental health services in high-risk regions, the promotion of lightweight AI intervention tools in low-resource settings, and substantial improvement in public health fund utilization efficiency (10). A core practical principle here is that AI tools should support, not replace, frontline MCH workers, with automated risk stratification to prioritize high-risk women for in-person care, optimizing limited human resources in low-resource settings.

Third, supporting the establishment of a multidisciplinary collaborative management platform. AI can facilitate secure data sharing across MCH institutions, mental health care facilities, and community health service organizations, enabling automated early warning, bidirectional referral, and full-process follow-up management for high-risk individuals, addressing the critical cross-sector collaboration gap in traditional systems (11).

## 4 Controversies, limitations and ethical challenges of AI in PPD prevention and control

While recognizing AI's significant application value, it is critical to address its core controversies, real-world implementation barriers, and ethical challenges in PPD public health prevention and control, which is the central focus of our critical discussion.

First, the digital divide may further exacerbate maternal and infant health inequities, directly contradicting public health's core principle of health equity. AI tool use is heavily dependent on smartphone access, network coverage, and digital literacy; however, smartphone penetration among women in low-income countries is below 30%. Women in rural areas, those with low educational attainment, and ethnic minority women face substantial barriers to accessing AI services, which may widen PPD prevention and control gaps across population groups, creating a dilemma whereby “the more advanced the technology, the more pronounced health inequities become (12, 13)”.

Second, substantial risks exist in the security and privacy protection of sensitive maternal and infant data. PPD-related data encompasses highly sensitive information, including maternal mental health status, reproductive privacy, socioeconomic status, and infant health data. Data breaches and improper use during AI model training and deployment may result in discrimination against women in employment, insurance, and social welfare domains (9). Globally, there remains a lack of comprehensive, specialized regulatory frameworks for maternal and infant mental health data protection, as well as unified industry standards. Additionally, less than 5% of existing AI-powered PPD tools have received regulatory approval (e.g., FDA or CE marking) for clinical use, creating significant legal and safety risks for large-scale public health deployment.

Third, AI models have notable limitations in generalizability and interpretability, posing risks of misdiagnosis and missed diagnosis at the public health level. The “black box effect” inherent to AI models precludes them from providing interpretable medical evidence to support risk predictions, limiting full acceptance by public health decision-makers and clinicians. Most existing AI models are trained and validated in high-income country populations, with a 20–30% drop in prediction accuracy in low-income and ethnic minority populations, severely limiting their generalizability for global public health application (4). Accordingly, AI models cannot be used as the sole basis for population-level prevention and control decisions (14).

Fourth, AI cannot replace humanistic care and structural social interventions. PPD onset is closely linked to key social determinants of health, including inadequate social support, intimate partner violence, economic hardship, and insufficient reproductive health benefits. AI can only address technical barriers related to screening and intervention, and cannot fundamentally reduce population-level PPD risk. The core of public health prevention and control remains dependent on structural social policy interventions, including reproductive security protections, anti-domestic violence measures, and inclusive childcare services.

## 5 Summary

AI technology offers revolutionary solutions to overcome the core bottlenecks of traditional PPD public health prevention and control. It can effectively expand population-level screening coverage, improve intervention accessibility, and optimize resource allocation efficiency, advancing the upgrade of PPD prevention and control to a full-cycle, population-based model. However, it must be critically emphasized that AI is only a supportive tool for public health prevention and control, not a standalone solution. Its real-world effectiveness is conditional on addressing the digital divide, ensuring data privacy, improving model generalizability, and aligning with structural social policy interventions.

AI application in this field must strictly adhere to the core public health principles of health equity, ethical integrity, and population-centeredness. Going forward, AI development in PPD public health prevention and control should prioritize dynamic and precise public health resource allocation, the improvement of specialized regulations for MCH data privacy protection, and the enhancement of model training in local populations and cross-population validation. These efforts will ultimately serve to reduce the global disease burden of PPD and uphold equal mental health rights for all postpartum women.
